# The Genomic Variation and Differentially Expressed Genes on the 6P Chromosomes in Wheat–*Agropyron cristatum* Addition Lines 5113 and II-30-5 Confer Different Desirable Traits

**DOI:** 10.3390/ijms24087056

**Published:** 2023-04-11

**Authors:** Wenjing Yang, Haiming Han, Baojin Guo, Kai Qi, Jinpeng Zhang, Shenghui Zhou, Xinming Yang, Xiuquan Li, Yuqing Lu, Weihua Liu, Xu Liu, Lihui Li

**Affiliations:** Key Laboratory of Grain Crop Genetic Resources Evaluation and Utilization, Institute of Crop Sciences, Chinese Academy of Agricultural Sciences, Beijing 100081, China; ywj103628@163.com (W.Y.); hanhaiming@caas.cn (H.H.); guobaojin1991@126.com (B.G.); zhangjinpeng@caas.cn (J.Z.); zhoushenghui@caas.cn (S.Z.); yangxinming@caas.cn (X.Y.); lixiuquan@caas.cn (X.L.); luyuqing@caas.cn (Y.L.); liuweihua@caas.cn (W.L.)

**Keywords:** wheat, *Agropyron cristatum*, wheat–*A. cristatum* 6P addition lines, heading date, grain number per spike, thousand-grain weight, genome resequencing, gene expression

## Abstract

Wild relatives of wheat are essential gene pools for broadening the genetic basis of wheat. Chromosome rearrangements and genomic variation in alien chromosomes are widespread. Knowledge of the genetic variation between alien homologous chromosomes is valuable for discovering and utilizing alien genes. In this study, we found that 5113 and II-30-5, two wheat–*A. cristatum* 6P addition lines, exhibited considerable differences in heading date, grain number per spike, and grain weight. Genome resequencing and transcriptome analysis revealed significant differences in the 6P chromosomes of the two addition lines, including 143,511 single-nucleotide polymorphisms, 62,103 insertion/deletion polymorphisms, and 757 differentially expressed genes. Intriguingly, genomic variations were mainly distributed in the middle of the chromosome arms and the proximal centromere region. GO and KEGG analyses of the variant genes and differentially expressed genes showed the enrichment of genes involved in the circadian rhythm, carbon metabolism, carbon fixation, and lipid metabolism, suggesting that the differential genes on the 6P chromosome are closely related to the phenotypic differences. For example, the photosynthesis-related genes *PsbA*, *PsbT,* and *YCF48* were upregulated in II-30-5 compared with 5113. *ACS* and *FabG* are related to carbon fixation and fatty acid biosynthesis, respectively, and both carried modification variations and were upregulated in 5113 relative to II-30-5. Therefore, this study provides important guidance for cloning desirable genes from alien homologous chromosomes and for their effective utilization in wheat improvement.

## 1. Introduction

Wheat (*Triticum aestivum* L.) is one of the most important cereal crops in the world, providing about 20% of the total dietary calories and proteins worldwide. It is now the most widely cultivated cereal in the world, with more than 220 million ha planted and about 670 million tons produced annually [1]. Consequently, wheat is one of the most important crops for global food security. However, wheat improvement for high yield and quality to meet the demands of the increasing human population and climate change has become challenging, as wheat breeding has been bottlenecked by decreasing genetic diversity [2]. Transferring desirable genes from wild relatives into common wheat is an effective approach for broadening wheat genetic diversity [3]. Many alien genes have been transferred into common wheat through chromosome translocations from its wild relatives, such as *Secale cereale* [4], *Hordeum californicum* [5], *Thinopyrum* [6,7], and *Aegilops peregrina* [8,9]. The 1RS chromosome of rye has been successfully introduced into wheat through a rye–wheat 1RS.1BL translocation line, and its resistance genes *Pm8*, *Lr26*, and *Yr9* have been widely used in wheat breeding [4,10,11,12]. *Pm21* from the wild species *Haynaldia villosa* confers high resistance to wheat powdery mildew and has become one of the most highly effective genetic loci introgressed into wheat from wild species [13,14,15,16]. Wheat genome modification by developing more translocation lines and introgression lines will be highly valuable for wheat breeding in the future.

*Agropyron cristatum* (L.) (2*n* = 4*x* = 28; genome PPPP), a wild relative of wheat, possesses many desirable traits, such as high tiller number, high floret numbers and spikelet numbers, and strong stress resistance [17,18]. Successful wide hybridization between common wheat and *A. cristatum* was performed in the 1990s [17], contributing greatly to the innovation of wheat germplasm resources. Following several generations of backcrossing and selfing, a series of disomic addition lines were developed, which could be used as starting materials to produce novel wheat germplasms including translocation lines and introgression lines carrying desirable agronomic traits [17]. Introgression of the *A. cristatum* 1P chromosome reduces leaf size and plant height to improve the plant architecture of common wheat [19]. The *A. cristatum* 2P and 3P chromosomes carry genes for broad-spectrum immunity to leaf rust and high resistance to powdery mildew [20,21]. The *A. cristatum* 5P chromosome affects homologous chromosome pairing [22], and the *A. cristatum* 6P chromosome confers many high-yield related traits to common wheat, such as multiple florets, higher grain number per spike (GNS), and multiple fertile tiller numbers [23,24,25]. Wheat–*A. cristatum* 6P disomic addition lines have been used to produce numerous derivative lines, some of which act as the fundamental material to improve yield-related traits in wheat [24,25,26,27]. The *A. cristatum* 7P chromosome can stably and significantly increase the grain weight in wheat [28]. *A. cristatum* constitutes useful germplasm for identifying excellent genes to enhance the genetic diversity of wheat.

During long-term evolution, the chromosome composition of plant species is gradually fixed in a given karyotype [29]. When the balance is disturbed, such as through the addition of alien chromosomes, the karyotype tends to become unstable [29]. This kind of chromosome instability may induce structural rearrangement and genome modification, which eventually lead to phenotypic variation [30,31]. Rearrangement of alien chromosomes in wheat–alien addition lines has been well documented, such as in wheat–rye, wheat–*Thinopyrum elongatum,* and wheat–*Leymus racemosus* addition lines [32,33,34]. When wheat–rye or wheat–*Leymus racemosus* addition lines were produced, several rearranged chromosomes were observed. Some were derived from a simple rearrangement, such as a Robertsonian translocation or misdivision, whereas others originated from more complex rearrangements [29,34]. The most unstable lines in the wheat–rye disomic addition lines were those with 2R and 4R additions [29]. Wheat–*A. cristatum* 6P disomic addition lines also underwent obvious rearrangements. Lines with different types of rearranged 6P chromosomes exhibited different phenotypes. For example, the 6P_I_-type addition lines 4844-12 and 5113 carried genes conferring high GNS and resistance to powdery mildew, while the 6P_II_-type addition line 5106 exhibited high grain weight [35]. Such chromosome rearrangements provide more variation for crop germplasm resource innovation.

Here, we identified two wheat–*A. cristatum* 6P addition lines, 5113 [26] and II-30-5 [36], containing different 6P chromosomes, which exhibited considerable differences in heading date, GNS, and thousand-grain weight, that is, late and early heading, high and low GNS, and low and high thousand-grain weight, respectively. To further reveal the relationship between chromosome differences and traits, we compared the two wheat–*A. cristatum* 6P addition lines at the genome and transcriptome levels. The sequence differences and gene expression differences explained the molecular basis for such a large phenotypic difference between the two lines. This study will provide important guidance for the cloning and utilization of the *A. cristatum* 6P genes in wheat breeding and provide a valuable reference for the creation of new germplasm materials and the transfer of alien genes into the wheat genome.

## 2. Results

### 2.1. 5113 and II-30-5 Exhibited Significant Differences in Heading Date, GNS, and Thousand-Grain Weight

**Figure 1:**
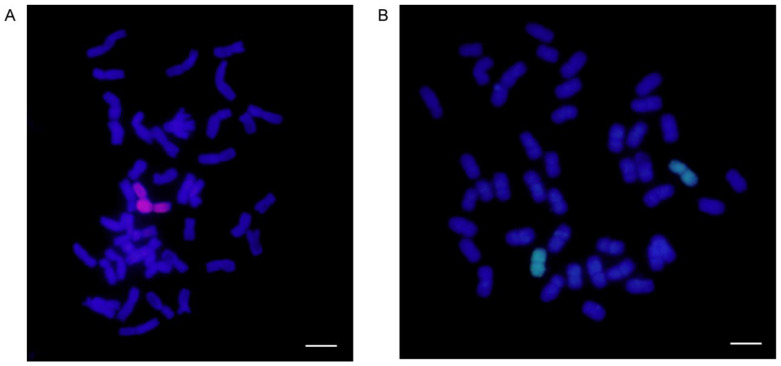
GISH results of wheat–*A. cristatum* 6P addition lines. (**A**) GISH of 5113; (**B**) GISH of II-30-5. The *A. cristatum* chromosomes are in red or green, while the wheat chromosomes are in blue. Scale bar, 10 μm.

**Figure 2:**
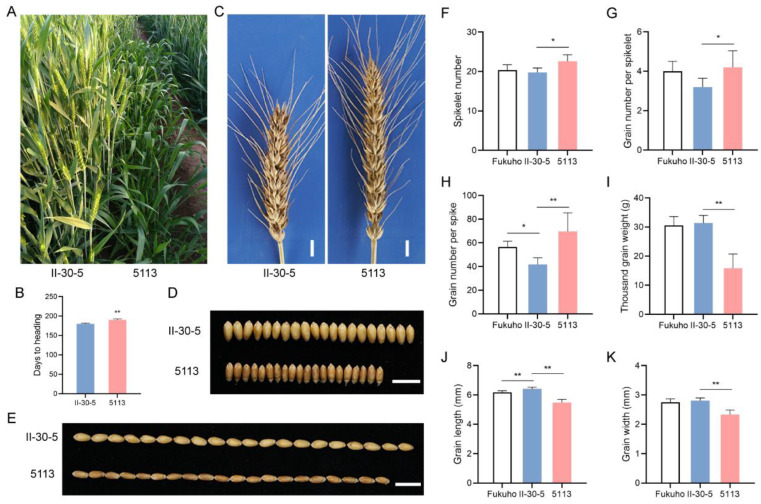
The differences between 5113 and II-30-5 in heading date, GNS, and thousand-grain weight in the field. (**A**) II-30-5 headed significantly earlier than 5113. (**B**) Days to heading of 5113 and II-30-5. (**C**) Comparison of the spikes of 5113 and II-30-5 at maturity. Bar = 1 cm. (**D**) Comparison of the grain widths of 5113 and II-30-5. Bar = 1 cm. (**E**) Comparison of the grain length of 5113 and II-30-5. Bar = 1 cm. (**F**) Spike number of 5113 and II-30-5. (**G**) Grain number per spikelet of 5113 and II-30-5. (**H**) Grain number per spike of 5113 and II-30-5. (**I**) Thousand-grain weight of 5113 and II-30-5. (**J**) Grain width of 5113 and II-30-5. (**K**) Grain length of 5113 and II-30-5. Error bars represent the SD of three biological replicates. Asterisks indicate statistically significant differences between addition lines and controls: *, *p* < 0.05; **, *p* < 0.01.

We initially identified two wheat–*A. cristatum* 6P addition lines, II-30-5 and 5113, that share the same recipient parent Fukuhokomugi (Fukuho) but carry different 6P chromosomes, which was confirmed by genome in situ hybridization (GISH). The results indicated that 5113 and II-30-5 both had two chromosomes from *A. cristatum* and 42 chromosomes from wheat (Figure 1). To identify the genetic effects of the two addition lines, the phenotype was evaluated in Xinxiang, Henan Province. Field phenotypic observations showed that II-30-5 and 5113 conferred significant differences in heading date, GNS, and thousand-grain weight. On average, II-30-5 headed 10 days earlier than 5113 (Figure 2A,B) and increased the thousand-grain weight by 16.6 g (Figure 2I) (Table S2). II-30-5 seeds, which appeared larger and paler, were clearly distinguished from 5113 seeds (Figure 2D,E). The grain width and grain length of II-30-5 were 0.59 mm and 0.44 mm higher, respectively, than those of 5113 (Figure 2J,K) (Table S2). Conversely, the GNS of 5113 was 28.0 higher than that of II-30-5 under field conditions (Figure 2C,H) (Table S1). The increased GNS in 5113 was mainly caused by the increased spikelet number per spike and grain number per spikelet (Figure 2F,G) (Table S1). These results indicated that II-30-5 had characteristics of early heading and high thousand-grain weight, while 5113 could significantly increase the GNS. Such a large phenotypic difference indicated genetic variation between the two addition lines.

### 2.2. The Genetic Variation of 5113 and II-30-5 at the Whole Genome Level

**Figure 3:**
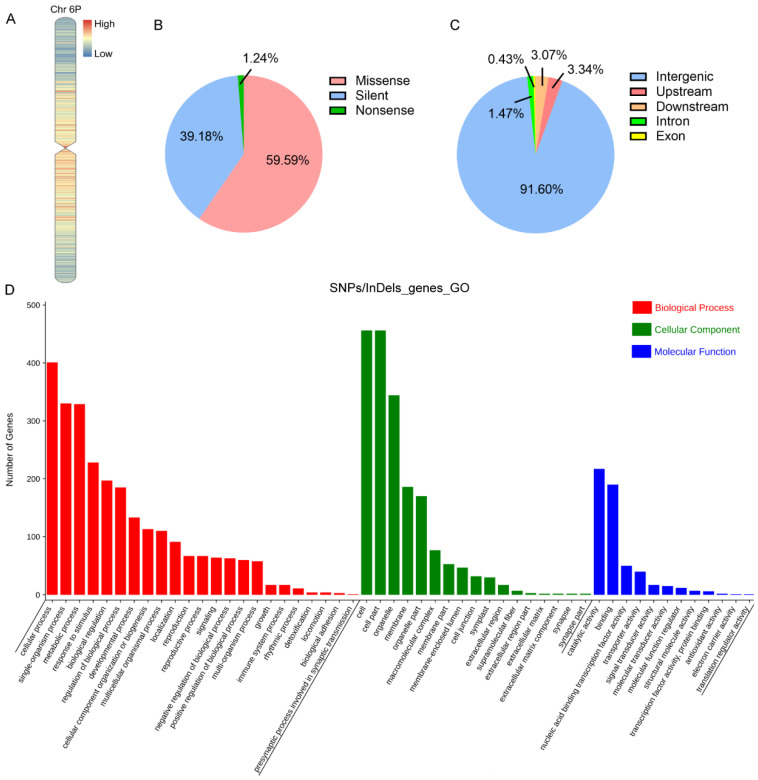
Genome resequencing analysis of 5113 and II-30-5. (**A**) Distribution of the SNPs and InDels in the 6P chromosomes of 5113 and II-30-5. (**B**) Summary of the effects of SNP variations on gene function. (**C**) Pie chart representing the distribution regions of SNPs/InDels in 5113 and II-30-5 6P variant genes. (**D**) GO enrichment of the SNP/InDel variant genes between 5113 and II-30-5.

To compare the genetic differences of 5113 and II-30-5 at the whole genome level, genome resequencing of the two addition lines was performed. By systematically comparing the resequencing data, 143,511 single-nucleotide polymorphisms (SNPs) and 62,103 insertion/deletion polymorphisms (InDels) were identified between the 6P chromosomes of 5113 and II-30-5, respectively. The SNPs and InDels were mainly distributed in the middle of the chromosome arms and proximal centromere region (Figure 3A). We then annotated the effects of SNPs on gene function and found that the majority of SNPs were missense mutations, accounting for 59.59% of all mutations (Figure 3B), followed by silent and nonsense mutations, accounting for 39.18% and 1.24%, respectively (Figure 3B). In terms of the distribution regions, SNPs/InDels were mainly distributed in intergenic regions, accounting for 91.60%, followed by regions upstream (3.34%) and downstream (3.07%) of genes, intron regions (1.47%), and exon regions (0.43%) (Figure 3C). According to the effect of mutations on genes, 99.57% of mutations were modification mutations, 0.19% were moderate mutations, 0.11% were low-impact mutations, and only 0.13% were high-impact mutations that included frameshift variants, splice acceptor or donor variants, and premature termination codon mutations. These results indicated that the phenotypic differences between the two 6P addition lines were mainly caused by high-impact gene mutations and gene expression differences from modification mutations. Without counting the SNPs/InDels in intergenic regions, a total of 3381 *A. cristatum* 6P genes carrying SNPs/InDels were identified (Table S3). Gene ontology (GO) enrichment analysis of these 3381 genes revealed that the circadian rhythm process, developmental process, metabolic process, and reproductive process were enriched (Figure 3D). The Kyoto Encyclopedia of Genes and Genomes (KEGG) also showed that the circadian rhythm pathway was enriched (Figure S1, Table S4).

### 2.3. RNA-Seq Analysis of the Addition Lines 5113 and II-30-5

To globally explore the transcriptomic alterations in 5113 and II-30-5, we performed RNA-seq using leaves at the floret differentiation stage. In all, 5113 and II-30-5 provided 210,147,490 and 204,952,102 clean reads, respectively, after the quality filtering process (Table S6). The Q20 and Q30 percentages of 5113 and II-30-5 were greater than 96.62% and 91.62%, respectively (Table S6). At least 82.94% of the clean reads were mapped to the wheat reference genome (IWGSC RefSeq v2.1). After read counting and normalization, a total of 75,081 genes were detected in 5113 and II-30-5. Subsequently, the correlation based on fragments per kilobase of exon per million fragments mapped (FPKM) among the six samples indicated high quality and reproducibility (Figure S2).

**Figure 4:**
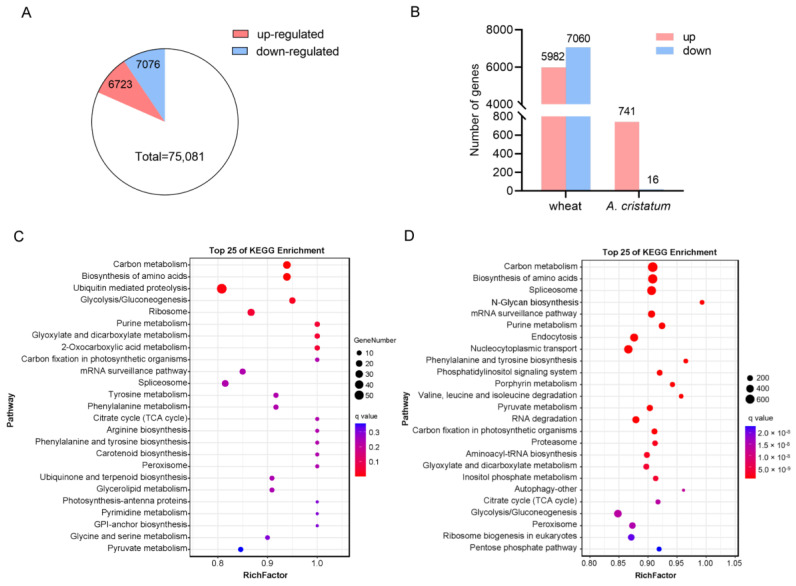
Comparison of DEGs between 5113 and II-30-5. (**A**) Pie chart of DEGs between 5113 and II-30-5. (**B**) Number of DEGs. (**C**) KEGG enrichment of *A. cristatum* DEGs. (**D**) KEGG enrichment of wheat DEGs.

Differentially expressed genes (DEGs) were identified by comparing gene expression between 5113 and II-30-5 (*p*-value < 0.05, absolute fold change > 1.0). A total of 13,799 DEGs comprising 6723 upregulated genes (741 *A. cristatum* genes and 5982 wheat genes) and 7076 downregulated genes (16 *A. cristatum* genes and 7060 wheat genes) were detected in II-30-5 relative to 5113 (Figure 4). To determine the biological process differences between 5113 and II-30-5, we subjected the DEGs to GO and KEGG enrichment analyses. The GO term associated with the circadian rhythm process was apparently enriched in both wheat and *A. cristatum* DEGs (Figure S3), which suggested that the early heading of II-30-5 was probably caused by the DEGs of the photoperiod pathway. Furthermore, the developmental process, metabolic process, and reproductive process were also enriched in both wheat and *A. cristatum* DEGs (Figure S3). For KEGG analysis, the modules for carbon metabolism, lipid metabolism, and carbon fixation in photosynthetic organisms were highly enriched in both wheat and *A. cristatum* DEGs (Figure 4C,D, Tables S7 and S8), explaining the large difference in seed development and thousand-grain weight of 5113 and II-30-5. Although the pathways enriched in DEGs in wheat and *A. cristatum* largely overlapped, the module of photosynthesis was only enriched in *A. cristatum* DEGs but not in wheat DEGs (Figure 4C), suggesting a large positive role for *A. cristatum* alien genes in the wheat background.

### 2.4. Changes in Photoperiod Pathway-Related Genes between 5113 and II-30-5

Among the sequence variation genes, the photoreceptor gene *cryptochrome 2* (*CRY2*) [37] (*Ac6P01G324000*) was found to contain seven SNPs and two InDels in the upstream and downstream regions of the gene (Table S5). CONSTANS (CO) plays important roles in the photoperiodic response of many plants [38]. Here, *CONSTANS-like 4* (*COL4*) (*Ac6P01G316300*) contained a one-SNP difference in downstream regions of the gene between the two addition lines (Table S5). Both *CRY2* and *CO* genes were modifying mutations. *Flowering locus T* (*FT*) is an integrator in plant flowering regulation [39]. Three *FT*-related genes were found to have sequence variation between the two addition lines, including two moderate missense variants in *Ac6P01G028100*, two moderate intron variants in *Ac6P01G052300*, and two SNP differences in the upstream and downstream regions of *Ac6P01G022700* (Table S5).

**Figure 5:**
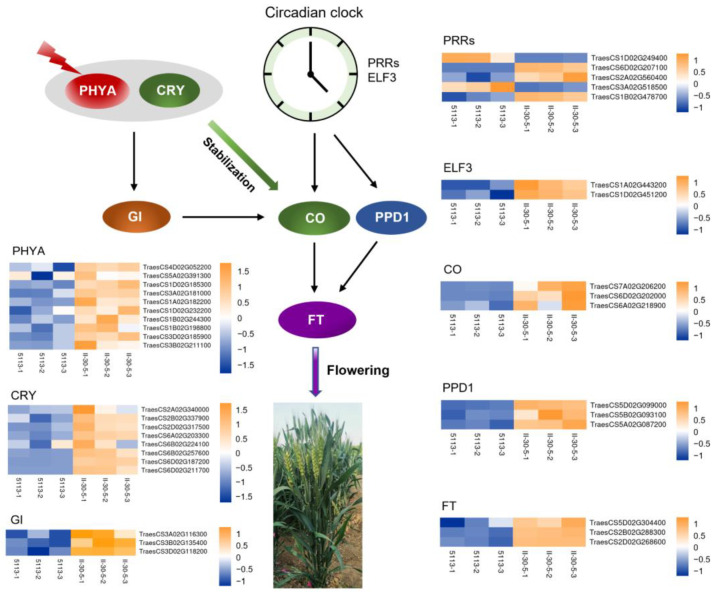
Schematic diagram of the flowering regulation pathway involving DEGs in the addition lines 5113 and II-30-5.

**Figure 6:**
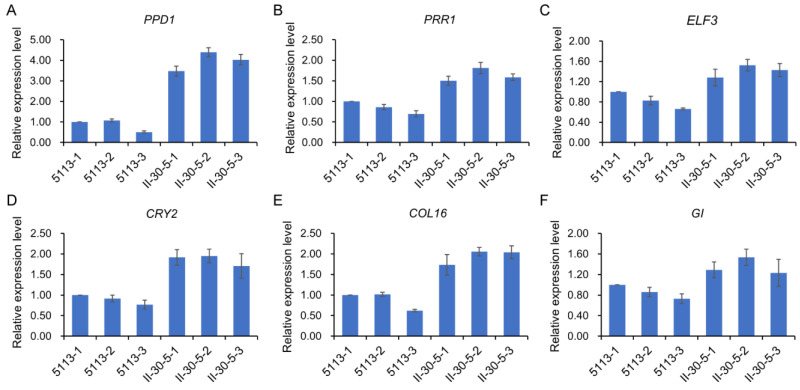
Verification of the expression levels of photoperiod pathway related genes. (**A**–**F**) Relative expression levels of the photoperiod gene *PPD1* (**A**), *PRR1* (**B**) *ELF3* (**C**) *CRY2* (**D**) *COL16* (**E**) and *GI* (**F**) in 5113 and II-30-5 plants. The *TaActin* was used as a housekeeping gene to calibrate the expression levels of genes.

In addition to sequence variation, photoperiod pathway-related genes were significantly differentially expressed between 5113 and II-30-5, such as the *PSEUDO RESPONSE REGULATOR* (*PRR*) family genes (Figure 5), which serve as a major component of the circadian clock [40]. *ELF3* (*EARLY FLOWERING3*) and its homologues [41] exhibited higher expression in II-30-5 than in 5113 (Figure 5). These circadian clock genes have been reported to affect the abundance of *CO* mRNA and to stabilize the CO protein, ultimately causing activation of *FT* transcription [42]. Here, the transcript levels of the *CO* and *FT* genes were increased in II-30-5 relative to 5113. At the same time, *phytochrome A* (*PHYA*) [43], *CRY2* [44], and *GIGANTEA* (*GI*) [38] were also upregulated in II-30-5 (Figure 5), all of which are involved in CO protein stabilization. The expression levels of these circadian clock genes were verified via qRT–PCR (Figure 6, Figure S4). These results at least partially explained the regulatory pathway of the early heading phenotype of II-30-5, and a regulatory pathway for early heading in II-30-5 was established (Figure 5).

### 2.5. Changes in Spike-Development-Related Genes between 5113 and II-30-5

MADS-box and homeobox domain genes are associated with floral organ development and meristem certainty, respectively [45,46]. Here, several MADS-box genes were found to contain modification mutations in upstream or downstream sequences (Table S5). Four homeobox domain genes were found to contain modification mutations in upstream or downstream regions and two moderate intron mutations (Table S5). Both classes of genes showed significantly differential expression between 5113 and II-30-5. The differences in the sequence and the expression of these genes may be related to the large differences in GNS.

**Figure 7:**
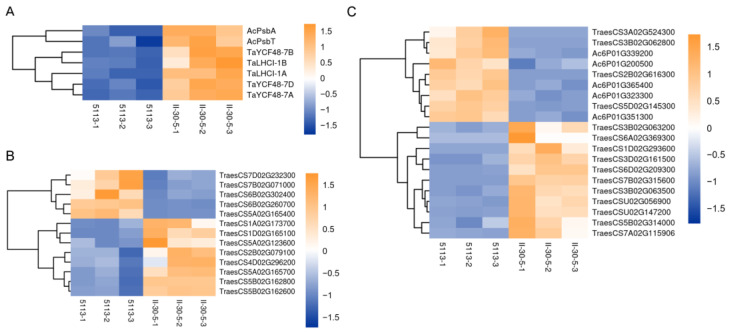
Heatmap of DEGs related to photosynthesis, carbon metabolism, and lipid metabolism between 5113 and II-30-5. (**A**) Differential expression of photosynthesis-related genes; (**B**) carbon-metabolism-related genes; (**C**) lipid-metabolism-related genes.

Photosynthesis is highly correlated with spike development and grain filling [47,48]. Here, photosynthesis-related genes were significantly upregulated in II-30-5 relative to 5113, and these genes included *PsbA* (*Ac6P01G008700*), *PsbT* (*Ac6P01G206200*), photosystem I light-harvesting complex I (*LHCI*) (*TraesCS1A02G392000*, *TraesCS1B02G420100*), and photosynthesis system II assembly factor *YCF48* [49] (*TraesCS7A02G560200*, *TraesCS7B02G486500*, *TraesCS7D02G549100*) (Figure 7). *PsbA* has been reported to encode the photosystem II (PS II) core reaction center complex [50], and *PsbT* plays key roles in optimizing the electron acceptor complex of the acceptor side of PS II [51]. YCF48 is essential for the assembly of the photosystem I and II reaction centers [49,52]. The upregulation of these genes in II-30-5 led to the higher photosynthetic efficiency of II-30-5, which eventually led to an increase in thousand-grain weight.

Additionally, sequence variation and expression differences in some carbon fixation and fatty-acid-biosynthesis-related genes were also found (Table S5). *ACS* (*Ac6P01G248300*), encoding an acetyl-CoA synthetase in carbon fixation [53,54,55], contained a SNP mutation (downstream variant) between 5113 and II-30-5 and was significantly upregulated in 5113 (Table S5). *FabG* (*Ac6P01G121700*), encoding an enzyme associated with fatty acid biosynthesis [56,57], comprised one indel (downstream variant) and one SNP mutation (upstream variant) between 5113 and II-30-5, being also upregulated in 5113 relative to II-30-5 (Table S5). These results indicated that the phenotype may be affected by the combination of sequence variation and expression level changes.

## 3. Discussion

Differences in genome size, chromatin organization patterns, and cell cycle duration are well known to cause genomic conflicts in newly formed hybrids [58]. Moreover, genetic variations induced by chromosome restructuring and modification eventually cause phenotypic variation [32]. Wang et al. identified 122 types of chromosome rearrangements, including centromeric, telomeric, and intercalary chromosome translocations, producing novel genetic variability [59]. Work is in progress to produce a collection of common wheat lines that carry various rearrangements of individual chromosomes of rye and barley, which facilitate chromatin introgression and wheat breeding programs [60]. In this study, on the basis of the large phenotypic differences between the two addition lines, we speculated that the 6P chromosomes carried by the two addition lines may have undergone chromosome rearrangement. Previous reports identified six wheat–*A. cristatum* 6P addition lines that could be divided into four types by cluster analysis [35], indicating that the six addition lines were derived from different F_1_ hybrids or had undergone chromosome rearrangement. It is not clear whether the rearrangement occurred before hybridization or after the formation of the addition lines. Most chromosomal variation is generally thought to occur at the ends of chromosomes, and the chromosomes of eukaryotes have been reported to exhibit a great deal of polymorphism among different individuals [61]. Here, on the basis of the heatmap of the distribution of differential sites, the SNPs and InDels were mainly distributed in the middle of the chromosome arms and the proximal centromere region, in contrast to the findings in previous reports. The centromere is a constitutive structure of the chromosomes in eukaryotic species that is required for the separation of chromosomes and chromatids during meiosis and mitosis. Centromere studies are hindered by sequence complexity, especially for wheat with more than 85% repetitive DNA [62]. However, the publication of wheat sequencing and assembly information has promoted research on centromeres. According to a recent report, active centromeres repositioned and shifted frequently during wheat evolution and the centromeric chromatin was more open, with higher levels of gene expression but lower gene density [63]. The regions adjacent to active centromeres also have highly expressed genes [63]. Whether there is a correlation between the phenomenon of the active variations being concentrated in the middle of the chromosome arms and the proximal centromere region and evolution requires further study.

The distinct levels of genome rearrangements and genetic constitution led to different phenotypes among different individuals in a population [64]. In *Secale cereale*, three wheat–rye 1RS.1BL translocation lines showed different degrees of resistance to stripe rust, which suggested that the diversity of resistance genes for wheat stripe rust exists in rye [32]. The genetic similarity indices for 1RS chromosomes of different translocation or substitution lines varied broadly from 0.44 to 0.81, also demonstrating great genetic diversity within 1RS chromosome arms, which may carry untapped variations that could potentially be used for wheat improvement [32]. Knowledge of the genetic diversity of different 6P chromosomes is helpful for creating more germplasm resources and improving the utilization efficiency of *A. cristatum* alien genes in wheat. In this study, on the basis of genome resequencing, high dimensional precision, and rich genomic variation information were revealed between the two investigated addition lines, and a total of 143,511 SNPs and 62,103 InDels were identified between the 6P chromosomes of 5113 and II-30-5. This kind of DNA sequence variation of individuals within the same species is widespread, and only a few sequence differences can cause phenotypic changes [65]. These sequence differences are the intrinsic reasons for the physiological and biochemical phenotypic variations among different individuals and act as one of the power sources for biological evolution [66]. In this study, 99.57% of mutations were modification mutations, and only 0.13% were high-impact mutations, indicating that the phenotypic differences between the two 6P addition lines were mainly caused by gene expression differences from modification mutations and high-impact gene mutations. Among the high-impact mutation genes, there are many genes with uncharacterized functions (Table S9), which will provide greater potential for the exploration and utilization of alien genes from *A. cristatum*.

With the accumulation of resequencing data for wheat materials, the characteristics and rules of genome sequence variation among wild wheat resources will be further revealed. Resequencing of 145 landmark cultivars revealed asymmetric subgenome selection and strong founder genotype effects on wheat breeding in China [67]. Whole-genome resequencing of 92 isolates of *Fusarium culmorum* resulted in the identification of 130,389 high-quality SNPs that were used for the first genome-wide association study in this phytopathogenic fungus [68]. Whole-genome resequencing of 968 bread wheat and its progenitors helped establish the Wheat Genome Variation Database (WGVD), an integrated web database including genomic variations and selective signatures [69]. Effective analysis and utilization of abundant genomic variation information will be helpful in elucidating the gene functions of key traits, assisting wheat breeders in utilizing excellent alien gene regions, and improving the efficiency of wheat variety breeding. The transcriptome approach quickly and comprehensively provides almost all the gene information of a species in a certain state and allows rapid identification of the gene regulatory networks and functional genes of important agronomic traits [70]. Previous work identified an *OsDREB1C* by comparing maize and rice transcriptomes and metabolomes, which was elucidated to boost grain yields and shorten the growth duration of rice [71]. By comparing the DEGs, researchers found that the expression of rice *OsPALs* is significantly induced by brown planthopper feeding. Knockdown or overexpression of *OsPALs* can significantly reduce or enhance brown planthopper resistance, respectively [72]. From this study, the combination of genome resequencing and transcriptome analysis of the two wheat–*A. cristatum* addition lines will provide important guidance for the cloning and utilization of the *A. cristatum* 6P genes in wheat breeding.

Collectively, our study provides the genetic effects, detailed sequence variation, and gene expression differences of the two 6P chromosomes, which could improve the utilization of *A. cristatum* alien chromosomes in wheat breeding and facilitate the identification of *A. cristatum* genes associated with phenotypic changes and the study of the corresponding mechanisms. The addition of the two 6P chromosomes resulted in completely different phenotypes, indicating that different karyotype environments could cause different chromosome rearrangements and sequence variation. Although a large number of variation sites have been found, the finding of the sites directly associated with a given phenotype still faces great challenges. The effect of sequence variation on gene expression also needs to be further explored. We believe that our study will help broaden the genetic basis of wheat and promote the mining of functional genes.

## 4. Materials and Methods

### 4.1. Plant Materials and Growth Conditions

The common wheat cultivar Fukuho (2*n* = 6*x* = 42, AABBDD) and the two wheat–*A. cristatum* 6P addition lines 5113 and II-30-5 (2*n* = 42 + 2) were kept by our laboratory. The plants used for RNA-seq were cultivated in a greenhouse at 23 °C and 70% relative humidity under a 16 h light and 8 h dark photoperiod. The Fukuho, II-30-5, and 5113 used for field phenotyping were grown under normal water and fertilization conditions in Henan Province, P. R. China. All the plants were planted in 2 m rows and spaced 0.3 m apart. In each row, 20 seeds were sown. Agronomic traits, including spikelet number, grain number per spikelet, GNS, thousand-grain weight, grain width, and grain length were evaluated at maturity. The heading date of the addition lines was evaluated as days from the sowing date to the date when approximately 50% of the spikes per line were visible. It took about 180–190 days from the sowing date to the heading date. Statistical analysis was performed using GraphPad Prism 8 software (GraphPad, San Diego, CA, USA). Differences between the addition lines and controls were analyzed by one-way ANOVA. *p*-values < 0.05 were considered significant. The sample size is indicated in the figure legends.

### 4.2. GISH for Wheat–A. cristatum 6P Addition Lines

GISH was performed in root tip cells to identify the 6P addition chromosomes as described by Han et al. [73]. *A. cristatum* genomic DNA was used as a probe, and Fukuho genomic DNA was used as a blocker to detect *A. cristatum* chromosomes in the wheat background. The oligonucleotide probes were synthesized by Shanghai Sangon Biotech Company (Shanghai, China). Images were captured using an Olympus Zeiss AX10 microscope (Olympus Corporation, Tokyo, Japan) equipped with a charge-coupled device (CCD) (Diagnostic Institute, Sterling Height, MI, USA) camera.

### 4.3. Genome Resequencing of the Two Addition Lines

The two wheat–*A. cristatum* 6P addition lines 5113 and II-30-5 used for resequencing were planted in a greenhouse. The leaves in the seedling stage were collected for DNA extraction and library preparation. The DNA libraries were generated using MGIEasy DNA library preparation kits following the manufacturer’s recommendations, and the libraries were sequenced using the BGISEQ-500 platform with a paired-end read length of 150 bp. The clean reads were mapped to the integrated reference sequences of Chinese Spring RefSeqv2.1 using BWA-MEM (https://github.com/kaist-ina/BWA-MEME/, accessed on 19 December 2022) [74], and the sequencing coverage of reads to the reference genome was calculated by BEDTools (https://github.com/arq5x/bedtools2, accessed on 27 December 2022) [75]. SAMtools mpileup was used to detect the SNPs and InDels (https://github.com/samtools/samtools/releases/tag/1.11, accessed on 2 February 2023) [76]. The distribution of SNPs and InDels between the two addition lines was obtained by the R package RIdeogram (https://CRAN.R-project.org/package=RIdeogram, accessed on 4 February 2023) [77].

### 4.4. RNA Extraction

Leaf tissues were collected from II-30-5 and 5113 in the floret differentiation stage without any treatment. The samples were separated and then dropped immediately into liquid nitrogen and stored at −80 °C for RNA extraction. Total RNA was extracted using the plant total RNA extraction kit (ZOMANBIO, Beijing, China) according to the manufacturer’s instructions. Total RNA was reverse transcribed into cDNA for qRT–PCR using a Reverse Transcriptase Kit (ZOMANBIO, Beijing, China).

### 4.5. RNA Sequencing and Transcriptome Analysis

The RNA integrity was assessed using the RNA Nano 6000 Assay Kit of the Bioanalyzer 2100 system (Agilent Technologies, Santa Clara, CA, USA). RNA-seq of the resulting 12 libraries was conducted on an Illumina HiSeq 2500 sequencing platform at Novogene (Novogene, Beijing, China). DESeq2 was used to analyze differential expression (https://bioconductor.org/packages/release/bioc/html/DESeq2.html, accessed on 2 May 2022) [78]. Genes with an adjusted *p*-value < 0.05 and absolute fold change (FC) ≥ 1 were considered differentially expressed between 5113 and II-30-5. KEGG functional annotation of DEGs was implemented by Eggnog Mapper (http://eggnog-mapper.embl.de/, accessed on 9 May 2022) [79]. GO and KEGG analyses were performed using the OmicShare tools (https://www.om-icshare.com/tools, accessed on 22 August 2022). The heatmaps and Venn diagrams were also constructed using the OmicShare tools (accessed on 22 August 2022).

### 4.6. qRT–PCR

To verify the RNA-seq data, eight *A. cristatum* DEGs were selected for qRT–PCR. The qRT–PCR primers were designed using Primer Premier 5 software. qRT–PCR was performed using a TB Green^®®^ Premix Ex Taq™ II (Takara, Osaka, Japan) kit in a Step One Plus Real-Time PCR System (Applied Biosystems, Carlsbad, CA, USA). Three technical replicates were set for each biological sample, and the relative quantification of gene expression was calculated by the 2^-∆∆Ct^ method. The wheat *Actin* (*TraesCS1B02G024500*) and *Tubulin* (*TUB*) (*TraesCS1D02G353100*) [80] genes were used as the housekeeping genes to calibrate the expression levels of genes. The primers used for qRT–PCR are listed in Table S10.

## 5. Conclusions

Overall, this study revealed the differences between 5113 and II-30-5 at the genome and transcriptome levels, thus explaining the phenotypic differences. Numerous SNPs and InDels led to gene sequence variation and modification of gene expression. The sequence variation and upregulated expression of flowering-promoting genes led to the early heading phenotype of II-30-5. The upregulated expression of photosynthesis-related genes in II-30-5 might have increased the photosynthetic efficiency of II-30-5, eventually resulting in an increase in thousand-grain weight. In addition, the modules for carbon metabolism and lipid metabolism were apparently enriched in both wheat and *A. cristatum* DEGs. *ACS* and *FabG* are related to carbon fixation and fatty acid biosynthesis, respectively, both of which carried modification variations and were upregulated in 5113 relative to II-30-5. These findings provide support for the identification of the gene regulatory networks and functional genes of key agronomic traits in wheat–*A. cristatum* 6P addition lines with complex genomes.

## Data Availability

The data for this study have been deposited in the NCBI database under BioProject accession number PRJNA953607 for genome resequencing and PRJNA910100 for RNA-seq.

## Supplementary Materials

The following supporting information can be downloaded at: https://www.mdpi.com/article/10.3390/ijms24087056/s1.
