# Supplementary material for: The Genomic Variation and Differentially Expressed Genes on the 6P Chromosomes in Wheat–Agropyron cristatum Addition Lines 5113 and II-30-5 Confer Different Desirable Traits
Source: Int J Mol Sci. 2023 Apr 11;24(8):7056. doi: 10.3390/ijms24087056 (PMC10139034; doi:10.3390/ijms24087056)
Supplement: Supplementary file 1 [file ijms-24-07056-s001.zip › Supplementary materials.pdf]

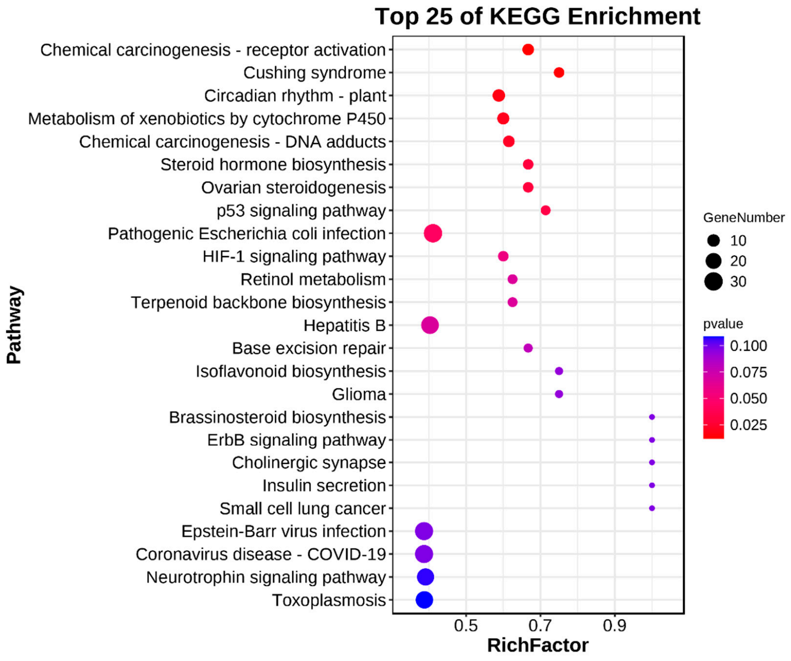

Figure S1. KEGG enrichment of 1860 *A. cristatum* 6P genes carrying SNPs/InDels.

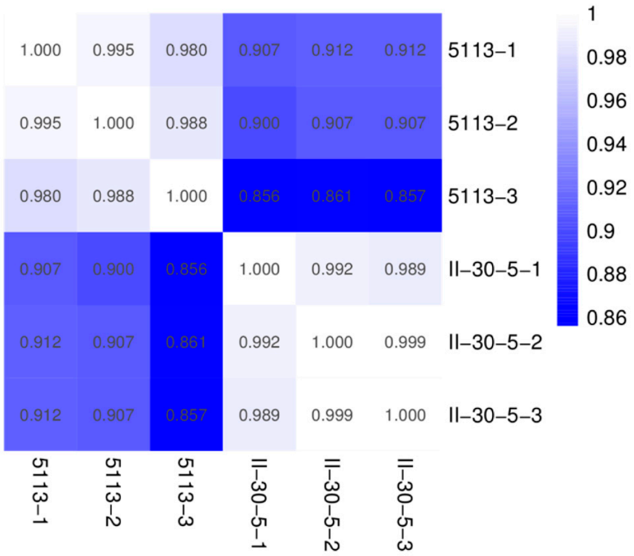

Figure S2. Correlation coefficients for each sample of addition lines 5113 and II-30-3.

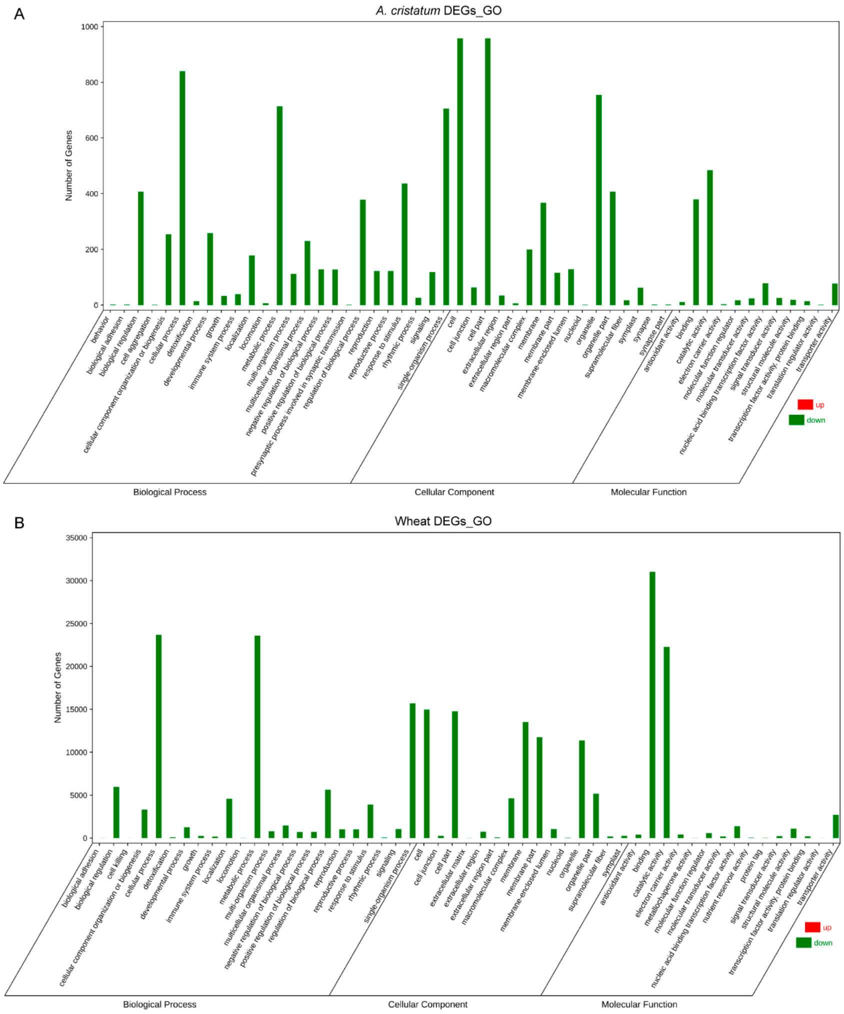

Figure S3. GO enrichment analyses of *A. cristatum* (A) and wheat (B) DEGs. (A) GO enrichment analyses of *A. cristatum* DEGs. (B) GO enrichment analyses of wheat DEGs.

Formatted: Font: Bold, Complex Script Font: Bold

Formatted: Font: Bold, Complex Script Font: Bold

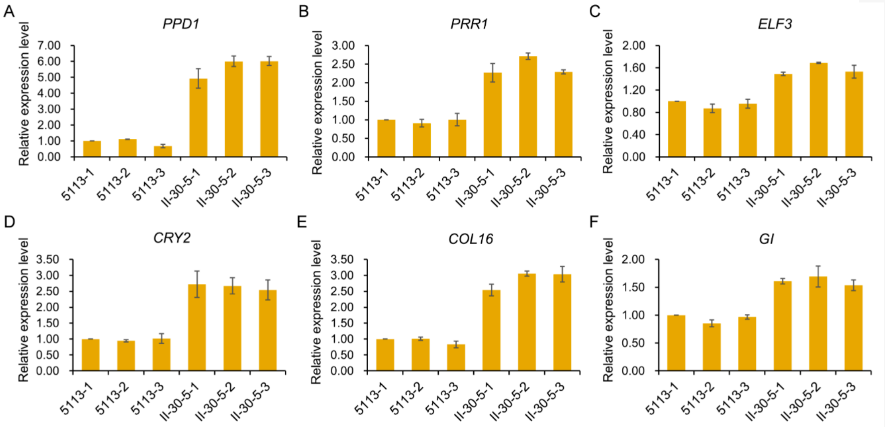

Figure S4. Verification of the expression levels of photoperiod pathway related genes. (**A-F**) Relative expression levels of the photoperiod gene *PPD1* (**A**), *PRR1* (**B**) *ELF3* (**C**) *CRY2* (**D**) *COL16* (**E**) and *GI* (**F**) in 5113 and II-30-5 plants. The *TaTUB* was used as a housekeeping gene to calibrate the expression levels of genes.

Formatted: Font color: Auto

Formatted: Font: Not Bold, Font color: Auto, Complex Script Font: Bold

Formatted: Font color: Auto, Complex Script Font: Bold

Table S1. Spikelet number per spike, grain number per spikelet and GNS of 5113 and II-30-5 under field conditions.

Year	Traits	Fukuho	5113	II-30-5
2020	Spikelet number per spike	20.33±0.79 (n=9)	22.60±0.79** (n=5)	19.80±0.89 (n=5)
	Grain number per spikelet	4.00±0.33 (n=9)	4.20±0.33** (n=5)	3.20±0.37 (n=5)
	GNS	56.56±5.00 (n=9)	69.80±5.00** (n=5)	41.80±5.67 (n=5)
2021	Spikelet number per spike	20.57±0.86 (n=7)	23.88±0.80** (n=6)	19.00±0.83 (n=8)
	Grain number per spikelet	4.14±0.37 (n=7)	4.38±0.35** (n=6)	3.33±0.36 (n=8)
	GNS	65.43±7.85 (n=7)	81.25±7.30** (n=6)	45.00±7.62 (n=8)
2022	Spikelet number per spike	19.00±0.48 (n=16)	23.00±0.48** (n=10)	20.70±0.53 (n=10)
	Grain number per spikelet	4.44±0.41 (n=16)	5.70±0.41** (n=10)	4.30±0.45 (n=10)
	GNS	68.13±6.15 (n=16)	100.50±6.15** (n=10)	58.70±6.70 (n=10)

n: Number of plants per line. GNS: Grain number per spike.
Data are presented as means ± S.E.s. Two-tailed Student's *t*-test; **P*<0.05, ***P*<0.01.

Table S2. Thousand-grain weight, grain length and grain width of 5113 and II-30-5 under field conditions.

Year	Traits	Fukuho	5113	II-30-5
2018	Thousand-grain weight	30.58±1.56 (n=9)	15.84±1.58 (n=15)	31.46±1.38** (n=14)
	Grain length	6.18±0.07 (n=9)	5.49±0.07 (n=15)	6.42±0.06** (n=14)
	Grain width	2.75±0.05 (n=9)	2.33±0.05 (n=15)	2.81±0.05** (n=14)
2019	Thousand-grain weight	34.73±1.18 (n=9)	23.92±1.13 (n=14)	40.37±1.06** (n=11)
	Grain length	6.76±0.05 (n=9)	6.13±0.05 (n=14)	6.57±0.05** (n=11)
	Grain width	3.11±0.04 (n=9)	2.65±0.04 (n=14)	3.24±0.04** (n=11)

n: Number of plants per line.
Data are presented as means ± S.E.s. Two-tailed Student's t-test; *P<0.05, **P<0.01.
